# Analysis of death causes of residents in poverty-stricken Areas in 2020: take Liangshan Yi Autonomous Prefecture in China as an example

**DOI:** 10.1186/s12889-022-12504-6

**Published:** 2022-01-13

**Authors:** Rujun Liao, Lin Hu, Qiang Liao, Tianyu Zhu, Haiqun Yang, Tao Zhang

**Affiliations:** 1 Sichuan Center for Disease Control and Prevention, 610041 Chengdu, Sichuan Province China; 2 Department of Epidemiology and Health Statistics, West China School of Public Health, West China Fourth Hospital, Sichuan University, Renmin South Road 3rd Section NO.16, Sichuan Province 610041 Chengdu, China; 3 Liangshan Prefecture Center for Disease Control and Prevention, 615000 Xichang, Sichuan Province China

**Keywords:** Cause of death, Mortality, Rank of death causes, Disease burden, Premature NCD mortality, Life expectancy, Capture-recapture method

## Abstract

**Background:**

Continuous surveillance of death can measure health status of the population, reflect social development of a region, thus promote health service development in the region and improve the health level of local residents. Liangshan Yi Autonomous Prefecture was a poverty-stricken region in Sichuan province, China. While at the end of 2020, as the announcement of its last seven former severely impoverished counties had shaken off poverty, Liangshan declared victory against poverty. Since it is well known that the mortality and cause of death structure will undergo some undesirable changes as the economy develops, this study aimed to reveal the distribution of deaths, as well as analyze the latest mortality and death causes distribution characteristics in Liangshan in 2020, so as to provide references for the decision-making on health policies and the distribution of health resources in global poverty-stricken areas.

**Methods:**

Liangshan carried out the investigation on underreporting deaths among population in its 11 counties in 2018, and combined with the partially available data from underreporting deaths investigation data in 2020 and the field experience, we have estimated the underreporting rates of death in 2020 using capture-recapture (CRC) method. The crude mortality rate, age-standardized mortality rate, proportion and rank of the death causes, potential years of life lost (PYLL), average years of life lost (AYLL), potential years of life lost rate (PYLLR), standardized potential years of life lost (SPYLL), premature mortality from non-communicable diseases (premature NCD mortality), life expectancy and cause-eliminated life expectancy were estimated and corrected.

**Results:**

In 2020, Liangshan reported a total of 16,850 deaths, with a crude mortality rate of 608.75/100,000 and an age-standardized mortality rate of 633.50/100,000. Male mortality was higher than female mortality, while 0-year-old mortality of men was lower than women’s. The former severely impoverished counties’ age-standardized mortality and 0-year-old mortality were higher than those of the non-impoverished counties. The main cause of death spectrum was noncommunicable diseases (NCDs), and the premature NCD mortality of four major NCDs were 14.26% for the overall population, 19.16% for men and 9.27% for women. In the overall population, the top five death causes were heart diseases (112.07/100,000), respiratory diseases (105.85/100,000), cerebrovascular diseases (87.03/100,000), malignant tumors (73.92/100,000) and injury (43.89/100,000). Injury (64,216.78 person years), malignant tumors (41,478.33 person years) and heart diseases (29,647.83 person years) had the greatest burden on residents in Liangshan, and at the same time, the burden of most death causes on men were greater than those on women. The life expectancy was 76.25 years for overall population, 72.92 years for men and 80.17 years for women, respectively, all higher than the global level (73.3, 70.8 and 75.9 years).

**Conclusions:**

Taking Liangshan in China as an example, this study analyzed the latest death situation in poverty-stricken areas, and proposed suggestions on the formulation of health policies in other poverty-stricken areas both at home and abroad.

**Supplementary Information:**

The online version contains supplementary material available at 10.1186/s12889-022-12504-6.

## Background

The death information of local residents is the reference to evaluate regional health level, determine the key intervention diseases, optimize the allocation of medical resources, and formulate relevant policies and programs [1]. Therefore, reporting death information in the system timely, monitoring death cases among the population continuously and analyzing death data regularly can measure health status of the population, reflect the health, economic level and cultural development of a region, thus promote development of health in the region and improve health level of residents [2, 3].

In 2017, there were 11 severely impoverished counties in Liangshan. At the same time, the prevalence of infectious diseases and noncommunicable diseases (NCDs) in Liangshan were at a high level, among which the HIV prevalence rate was one of the highest in China [4]. These health problems often led to poverty due to illness and return to poverty due to illness, which restricted the poverty alleviation work in Liangshan.

Under the strategic deployment of a well-off society in an all-round way, Liangshan announced at the end of 2020 that all the last 7 severely impoverished counties had shaken off poverty, winning the battle against poverty and heading to prosperity. The year of 2020 was the most important time for poverty alleviation in Liangshan and even in China. Health poverty alleviation is an important guarantee for poverty alleviation, and death surveillance is a key work in health poverty alleviation. Therefore, the study of death situation in Liangshan in such a key year will help consolidate poverty alleviation achievements.

However, with economy development, the living environment, lifestyle, values, psychological structure, behavior habits and other aspects of residents have also undergone drastic changes and conflicts with each other. Unbalanced economic and social development exposes deep-seated social problems, such as environmental deterioration, polarization between rich and poor, unbalanced social mentality, medical and health problems, etc. [5]. For example, globalization and economic development had promoted the transformation of diet structure in many countries, with refined sugar, refined fat, meat and oil as the main diet, which greatly increased the incidence of NCDs and reduces life expectancy [6]. In China, since the end of 1970s, with the reform and opening up, the incidence of type 2 diabetes had increased from less than 1% of the total population in 1980 to about 10% in 2008, becoming the global diabetes epidemic center [7]. Nauru, an independent nation established in 1968, had exploited a large number of phosphate deposits, making it used to have the highest per capita income in the world. Improved living conditions and reduced physical activity have induced obesity and diabetes [8]. In 1975, the prevalence of diabetes in Nauru was as high as 34.4%, ranking second in the world [9]. These studies show that with the improvement of economic conditions and lifestyle changes, the health status will undergo some undesirable changes, especially for NCDs such as diabetes, resulting in related deaths.

To understand the latest mortality and death causes distribution characteristics in this once deeply-impoverished area, this study used the data of death surveillance and the underreporting deaths investigation to analyze the distribution of deaths in Liangshan in 2020. Specifically, this study explored the differences of death status in different economic level counties, and compared the indicators including life expectancy and disease burden in different sexes. These efforts could not only be useful for consolidating the achievements of poverty alleviation in Liangshan, but also provide references for the formulation of health policies and the distribution of health resources in global poverty-stricken areas.

## Methods

### Death data collection

Firstly, to analyze death status in Liangshan in 2020, we collected the death surveillance data of 2020 from the cause of death registration and reporting information system of Liangshan Center for Disease Control and Prevention, with 16,850 death cases reported. Then, considering that substantial underreporting existed in the initial death data, Liangshan carried out an investigation on underreporting deaths in 2018 specially. Therefore, we also collected the death surveillance data from 2014 to 2018 and underreporting deaths investigation data in Liangshan to correct the underreporting.

### Population data collection

The population data came from the public security citizen household registration information system of Liangshan, which included the population data of different counties, sexes and age groups in 2020. The national census data of China in 2010 was taken as standard population.

### Quality control

#### Relevant document

In 1992, the Ministry of Health, the Ministry of Public Security and the Ministry of Civil Affairs in China jointly issued *the Notice on Using the Medical Certificate of Death and Strengthening the Statistics of Death Causes (Wei Tongfa (1992) No.1)* [10]. Subsequently, the Ministry of Health and the Chinese Center for Disease Control and Prevention successively issued *the Notice on the Implementation Plan of Death Surveillance in Medical Institutions at or above the County Level (Trial)*, *the National Work Standards for Disease Control and Prevention Institutions*, and *the National Work Standards for Death Surveillance in Disease Surveillance Systems*, which were used for the work of death cause registration and reporting [11]. Since 2013, in order to further standardize the surveillance work, the National Health and Family Planning Commission, the Ministry of Public Security and the Ministry of Civil Affairs jointly issued *the Notice on Further Regulating the Medical Certificate and Information Registration Management Work of Population Death*, requiring all localities to use the new version of *Medical Certificate (Inference) of Residents’ Death* to register residents’ deaths uniformly, and carry out information check work regularly [12]. These documents made death surveillance work smoothly and guaranteed data quality.

#### Guideline specification

According to the tenth revision of International Classification of Diseases (ICD-10), the underlying causes of death were determined and coded. To rank the death causes, this study divided the causes of death into 19 categories by referring to Appendix 4 Comparison Table of ICD-10 Coding for Diseases in Rank of Death Causes in China Death Surveillance Data Set 2019 [13]. At the same time, to study death status from different diseases category, the causes of death were grouped into three categories on the basis of the classification criteria of the World Health Organization [14]. The first category of diseases refers to infectious diseases, maternal-infant diseases and nutritional deficiency diseases, the second category of diseases refers to chronic non-communicable diseases, and the third category of diseases refers to injury.

#### The investigation of underreporting deaths

According to the requirements of the Chinese Center for Disease Control and Prevention and the Sichuan Center for Disease Control and Prevention, Liangshan carried out the investigation on underreporting deaths among population in its 11 counties in 2018, and recorded the underreporting cases into the all-cause death surveillance subsystem of the Chinese Information System for Disease Control and Prevention. In 2020, Liangshan started a new round of underreporting deaths investigation, which has not been totally completed so far. However, combined with the partially available data from underreporting deaths investigation data in 2020 and the field experience, it was estimated that the underreporting rates of death among different counties reduced by 16–20% in 2020 compared with that in 2018. The dramatic reduction was mainly due to the strictly management measures taken right after the outbreak of COVID-19. Specifically, according to the requirements of the central government, Liangshan has made a series of efforts to strengthen the localized healthcare management, demanding all the local authorities, line departments, employers and individuals should step up to their responsibilities to timely reporting of health events such as symptom, illness and death. As a consequence, the reduced death underreporting rate was further used to estimate the mortality and cause of death structure of Liangshan in 2020.

### Statistical method

The mortality was age-standardized by using China’s national population composition data of the sixth census in 2010. The crude mortality rate, age-standardized mortality rate, rank and proportion of death causes, potential years of life lost (PYLL), average years of life lost (AYLL), potential years of life lost rate (PYLLR), standardized potential years of life lost (SPYLL), premature NCD mortality, life expectancy and cause-eliminated life expectancy were calculated. An additional file shows the calculation method of each indicator (see Additional file 1).

Using capture-recapture (CRC) method [15], the underreporting rates of death of the overall population, men and women in different age groups, the underreporting rates of death of former severely impoverished counties and non-impoverished counties in different age groups in Liangshan during 2014-2018 were calculated. CRC method is a commonly used method to estimate the total mortality. Based on sampling theory, the total death number is estimated by two different ways of death data, namely, the investigation on underreporting deaths in Liangshan in 2018 and the death surveillance data of residents in Liangshan in 2014-2018. This study adopted the unbiased estimation formula proposed by Chapman and Wittes [16]:

$$N=\left[ {\frac{{\left( {{n_A}+1} \right)\left( {{n_B}+1} \right)}}{{\left( {{n_{11}}+1} \right)}}} \right] - 1,$$

$$R=(1 - \frac{{{n_A}}}{N})*100\% ,$$

where *N* is the estimated number of total death cases, *n*_*A*_ is the number of death cases recorded in death surveillance system, *n*_*B*_ is the number of death cases in the underreporting deaths investigation, *n*_11_ is the number of death cases recorded in both of the death surveillance system and underreporting deaths investigation, and *R* is the underreporting rates of death.

Then, the underreporting rates of death in 2020 for each county in Liangshan was calculated by multiplying the underreporting rates of death from 2014 to 2018 with its corresponding correction rate of 0.82(0.80-0.84). In addition, there were 13 death cases with unknown sex due to manual data input errors. However, given that the other information of them were complete, they were included for analyzing the death situation of overall population, while excluded in the analysis of different sexes. Finally, the results of this study reported in the following part were corrected for underreporting if not specified. All of the analysis work was conducted by Excel 2019 and R 4.0.3.

## Results

### Death of Liangshan Residents in 2020

#### Total death status of residents

In 2020, a total of 16,850 deaths were reported in Liangshan, with the crude mortality rate before correction being 316.06/100,000 and the age-standardized mortality rate before correction being 327.48/100,000. The mortality rate increased after being corrected for underreporting. The mortality rate of the overall population was 608.75/100,000, and the age-standardized mortality rate was 633.50/100,000. Among them, there were 10,239 male deaths, with a mortality rate of 720.60/100,000 and an age-standardized mortality rate of 761.92/100,000. There were 6,598 female deaths, with a crude mortality rate of 489.26/100,000 and an age-standardized mortality rate of 497.04/100,000 (see Table 1 in Additional file 2).

### Death status of residents by sex and age group

**Fig. 1:**
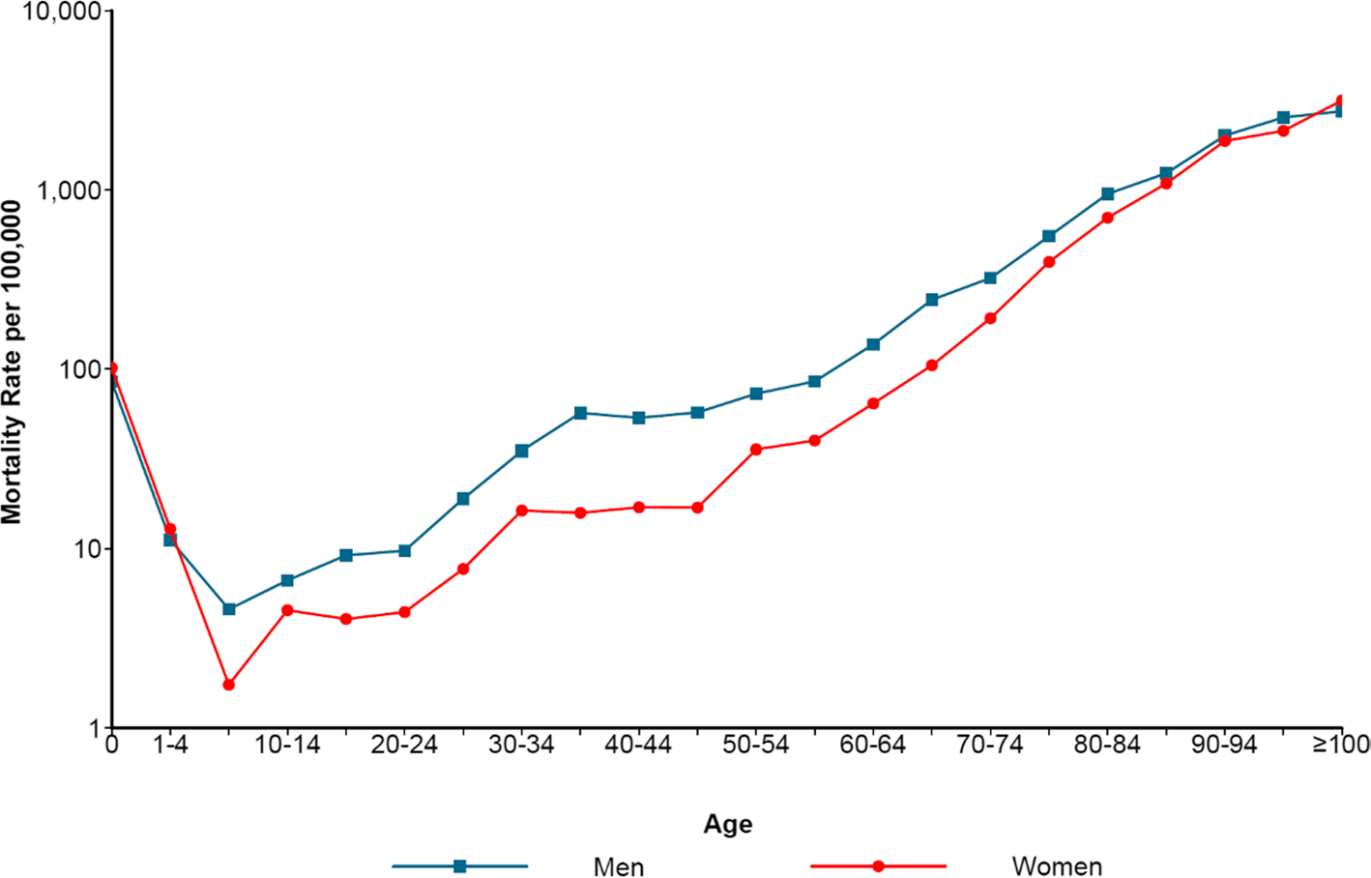
Mortality of different sexes and ages in Liangshan in 2020 (The blue line represents the men and the red line represents the women)

It can be seen from the semilogarithmic graph of mortality rate of different sexes and age groups (Fig. 1) that in different sexes in Liangshan, the age distribution of mortality rate was approximately V-shaped. The mortality rate in the 0-year-old group was high, which was 1,016.18/100,000 for women, higher than men (851.59/100,000). The mortality rate decreased rapidly before the age of 5, and reached the lowest point in the age group of 5-9 years for both of men and women, with the mortality rate being 45.94/100,000 and 17.44/100,000 respectively. After 9 years old, the mortality rate of men aged 35-39 was higher than that in the adjacent age groups, which was 568.74/100,000, and the mortality rate of women aged 30-34 was higher than that in the adjacent age groups, which was 163.50/100,000. Except for 0-4 years old and ≥100 years old, the mortality rates of men were higher than women’s (see Table 1 in Additional file 2).

### Death of residents in counties with different economic levels

**Fig. 2:**
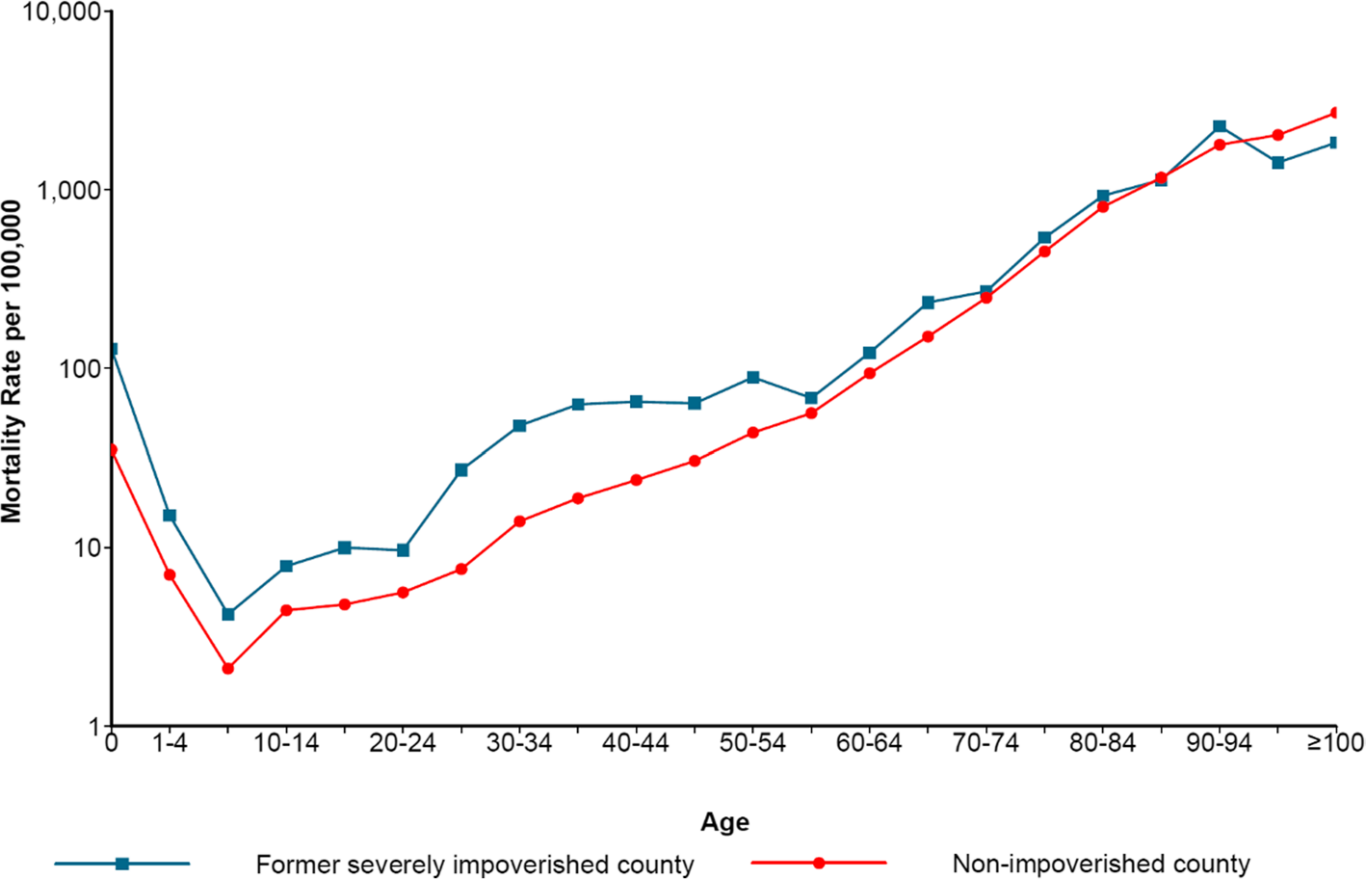
Age-specific mortality rate in counties at different economic levels in Liangshan in 2020 (The blue line represents the former severely impoverished counties and the red line represents the non-impoverished counties). The former severely impoverished counties refer to Puge county, Butuo county, Jinyang county, Zhaojue county, Xide county, Yuexi county and Meigu county, while the non-impoverished counties refer to the rest of Liangshan except the former severely impoverished counties

Similar to the above-mentioned distribution of sex and age mortality rate, in the semilogarithmic graph of mortality rate, the age distribution of mortality rate in former severely impoverished counties and non-impoverished counties in Liangshan was approximately V-shaped (Fig. 2). The crude mortality rate in former severely impoverished counties was 585.82/100,000, while in non-impoverished counties was 617.36/100,000. The mortality rate of 0-year-old group in former severely impoverished counties was much higher than that in the non-impoverished counties, which was 1,290.52/100,000 and 351.66/100,000 respectively. The mortality rate decreased at the age of 1, and reached the lowest point at the age of 5-9, which was 42.38/100,000 in former severely impoverished counties and 21.06/100,000 in non-impoverished counties. After 9 years old, the mortality rate showed a continuous upward trend. Except for 85-89 years old and ≥95 years old, the mortality rates in the former severely impoverished counties were higher than those in the non-impoverished counties. In addition, the age-standardized mortality rate of the former severely impoverished counties was 821.78/100,000, which was higher than 545.97/100,000 in non-impoverished counties (see Table 2 in Additional file 2).

### Demographic characteristics of dead residents and distribution of death places

In 2020, as shown in Table 1, the deaths of Liangshan residents were 10,239 (60.76%) and 6,598 (39.16%) for men and women, respectively. In the age proportion of the dead residents, the number of deaths in the age groups of 18-65, 66-79 and 80-99 were the highest, accounting for 32.85%, 32.42% and 30.99% respectively. The death toll from junior high school and below was the highest, with 15,965 (94.75%). Most of the dead residents were married, with a total of 11,515 (68.34%) 12,494 cases (74.15%) died at home, followed by medical and health institutions, with 3,095 cases (18.37%).

**Table 1:** Demographic characteristics of death residents in Liangshan in 2020

Demographic characteristic		Number of report cards	Proportion (%)
Sex	men	10,239	60.76
	women	6,598	39.16
	unknown	13	0.08
Age (years)	≤17	602	3.57
	18-65	5,536	32.85
	66-79	5,462	32.42
	80-99	5,222	30.99
	≥100	28	0.17
Education background	junior high school and below	15,965	94.75
	above junior high school	885	5.25
Marital status	divorce	166	0.99
	widowed	3,621	21.49
	unmarried	1,374	8.15
	married	11,515	68.34
	unknown	174	1.03
Place of death	at home	12,494	74.15
	medical and health care institution	3,095	18.37
	on the way to the hospital	290	1.72
	pension service organization	37	0.22
	other places	811	4.81
	unknown	123	0.73

### Death status of three categories of diseases among residents

**Fig. 3:**
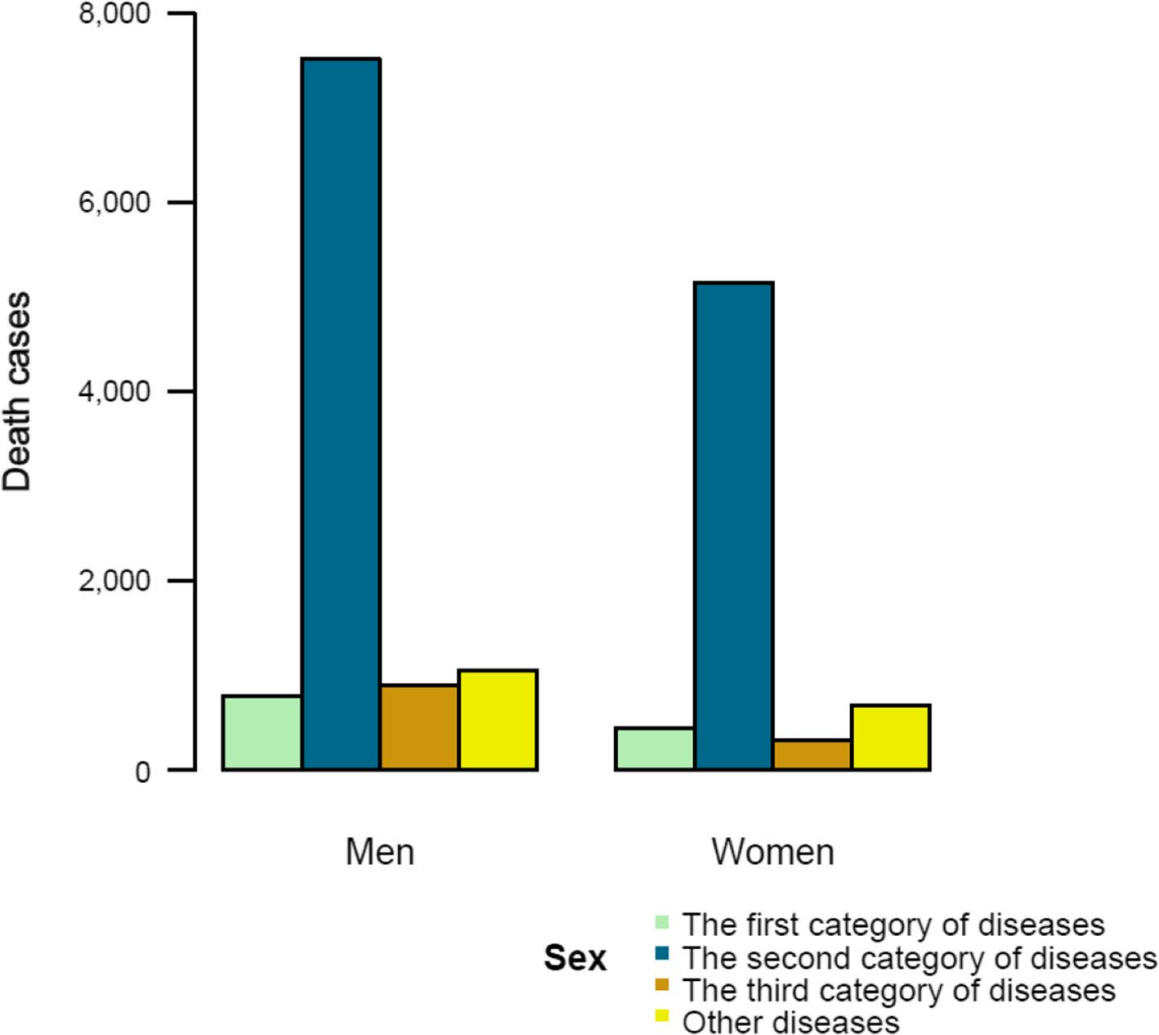
Death cases of three categories of diseases of different sexes in Liangshan in 2020 (The green bars represent the first category of diseases, the blue bars represent the second category of diseases, the brown bars represent the third category of diseases, and the yellow bars represent other diseases)

In 2020, the mortality rates of three categories of diseases in Liangshan were 44.44/100,000, 457.66/100,000, 43.89/100,000 respectively, and the second category of diseases, NCDs, had the highest proportion of 75.18%. Among the men and women, the mortality of NCDs were the highest, which were 528.75/100,000 (73.37%) and 381.67/100,000 (78.01%), respectively (see Table 3 in Additional file 2). The death cases and mortality rates of men were higher than those of women in all three categories of diseases (Fig. 3).

### The rank and proportion of death causes of residents

**Fig. 4:**
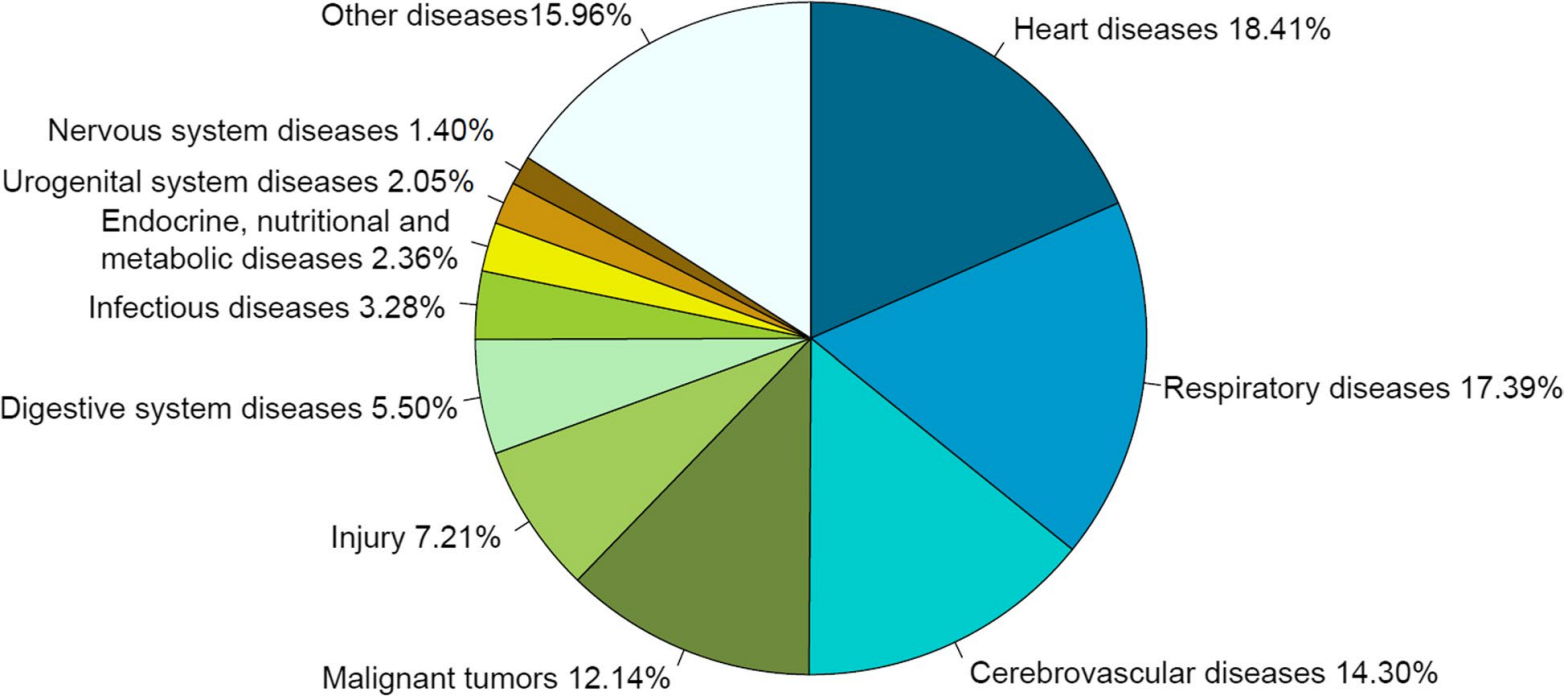
Proportion of the top ten death causes in Liangshan in 2020

**Fig. 5:**
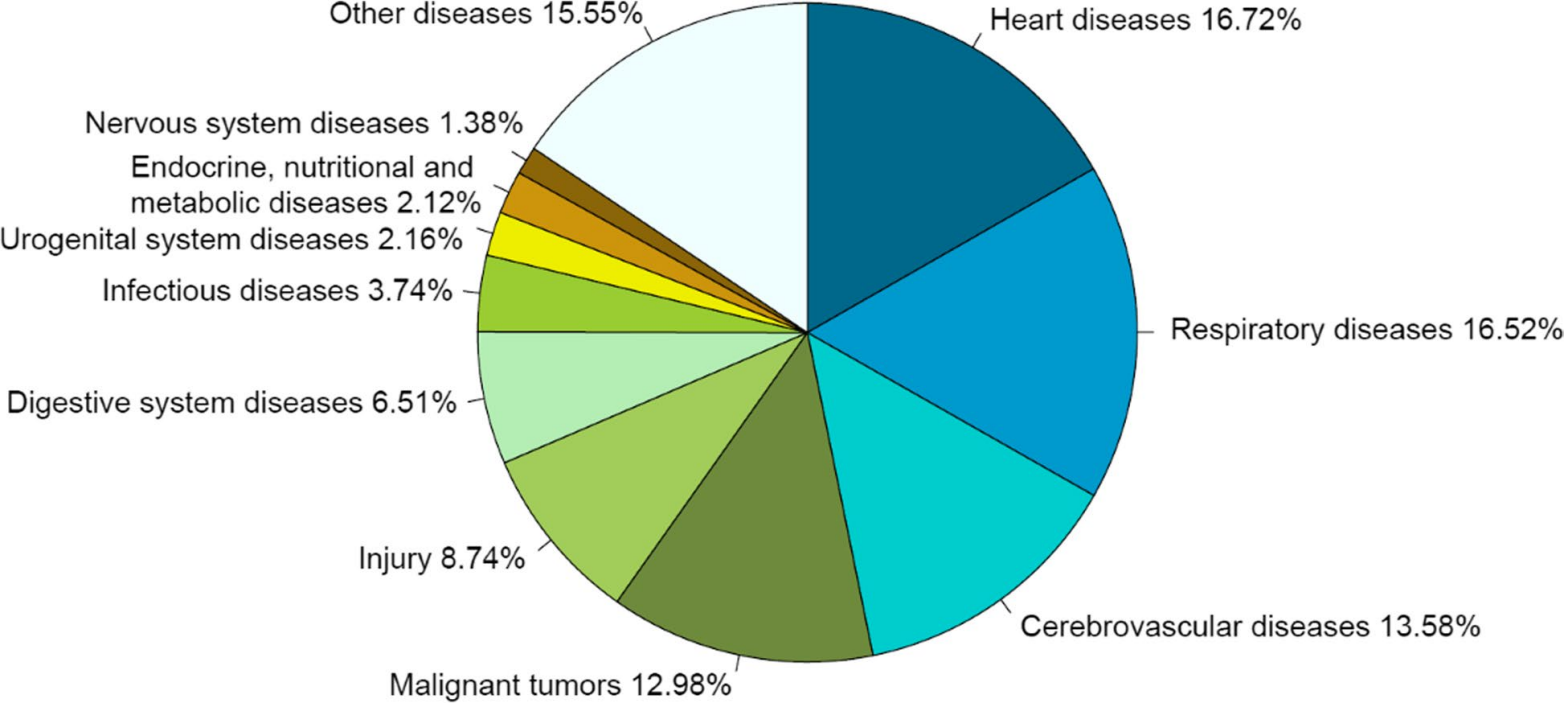
Proportion of the top ten death causes of men in Liangshan in 2020

**Fig. 6:**
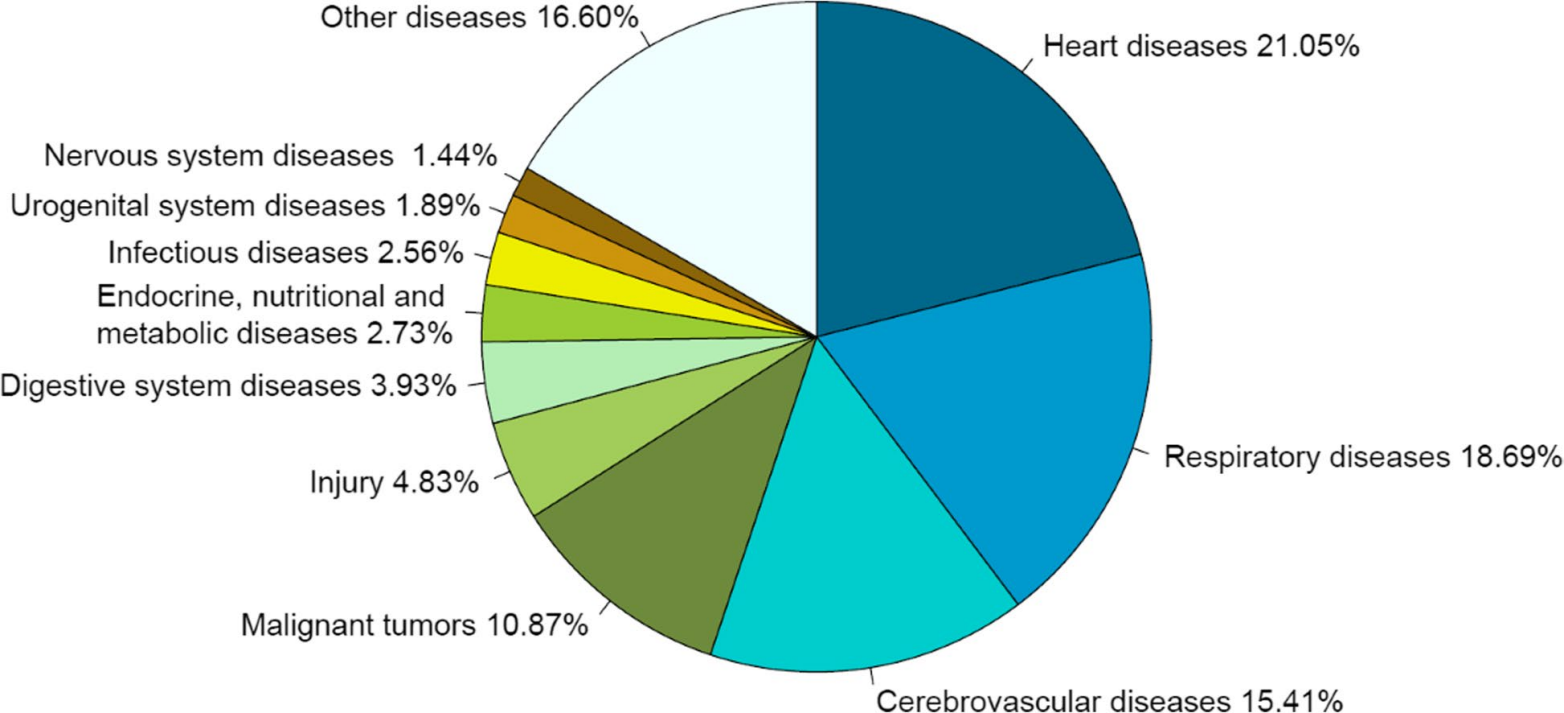
Proportion of the top ten death causes of women in Liangshan in 2020

In 2020, the top 10 death causes in Liangshan were heart diseases, respiratory diseases, cerebrovascular diseases, malignant tumors, injury, digestive system diseases, infectious diseases, endocrine, nutritional and metabolic diseases, urogenital system diseases and nervous system diseases (Fig. 4), accounting for 84.04% in total. The mortality of heart diseases was the highest, with the crude mortality, the age-standardized mortality, and the proportion were 112.07/100,000, 109.94/100,000, 18.41% respectively. The top 10 death causes among men and women were consistent with the death causes among the overall population, but the ranks of some diseases have changed (Figs. 5 and 6). Infectious diseases ranked the 7th among the overall population, but ranked the 8th among women. Endocrine, nutritional and metabolic diseases ranked the 8th in the overall population, but ranked the 9th in men and the 7th in women. Urogenital system diseases ranked the 9th in the overall population, but ranked the 8th in men. The top 10 death causes, heart diseases, respiratory diseases in men accounted for 84.45%, 16.72%, and 16.52%, while in women accounted for 83.40%, 21.05%, and 18.69% (see Table 4 in Additional file 2).

### AIDS death of residents

In 2020, AIDS accounted for 1.16% of all deaths in Liangshan, and 35.33% of deaths from infectious diseases. The crude mortality rate was 7.04/100,000, and 10.16/100,000 after age standardization. The crude mortality rate of men was higher than that of women, being 10.63/100,000 and 3.26/100,000, respectively.

### Analysis of disease burden of residents

#### Potential years life loss of all death causes

In 2020, the PYLL, AYLL, PYLLR and SPYLL caused by all deaths in Liangshan were 324,029.68, 9.96, 60.78‰ and 352,517.59 respectively. In men, PYLL was 226,791.64 person years, AYLL was 11.49 years/person. In women, PYLL was 95,347.49 person years, AYLL was 7.50 years/person. PYLL, AYLL, PYLLR and SPYLL caused by the death of male residents were all higher than those of female residents (see Table 2).

**Table 2:** Analysis of potential life loss of Liangshan residents in 2020 ^a^

Indicators	Overall population	Men	Women
PYLL (person years)	324,029.68	226,791.64	95,347.49
AYLL (years/person)	9.96	11.49	7.50
PYLLR (‰)	60.78	82.77	36.80
SPYLL (person years)	352,517.59	251,285.01	98,617.13

^a^ Note: The underreporting death was corrected for different age and sex groups

#### Potential years life loss of specific death cause

Compared with the death cause rank of residents, injury rose from the fifth place to the first place in the life loss rank, resulting in the largest PYLL of 64,216.78 person years, accounting for 19.82% of the total life loss, followed by malignant tumors, heart diseases, infectious diseases and cerebrovascular diseases, etc. The life loss of the top five death causes accounted for 59.17% of the total life loss of residents (see Table 3). Parasitic diseases caused the largest AYLL, which was 45.89 years/person, followed by obstetric diseases, congenital abnormality, injury and infectious diseases. The PYLLs caused by injury were the largest for men and women, which were 48,647.99 person years and 14,451.34 person years respectively. The AYLL caused by parasitic diseases was the largest in men, which was 43.92 years/person, and the AYLL caused by obstetric diseases was the largest in women, which was 41.93 years/person (see Table 5 in Additional file 2).

**Table 3:** Analysis of disease burden of residents in Liangshan in 2020^a^

Death cause	Overall population			
	PYLL (person years)	AYLL (years/person)	PYLLR (‰)	SPYLL (person years)
Injury	64,216.78	25.24	12.05	64,892.73
Malignant tumors	41,478.33	10.58	7.78	47,068.93
Heart diseases	29,647.83	5.11	5.56	34,073.83
Infectious diseases	29,611.09	24.68	5.55	32,418.50
Cerebrovascular diseases	26,770.14	5.92	5.02	31,089.60
Respiratory diseases	23,290.79	4.23	4.37	23,968.20
Digestive system diseases	19,239.74	10.76	3.61	23,450.41
Nervous system diseases	6,839.03	14.63	1.28	6,606.49
Urogenital system diseases	4,504.41	6.90	0.84	5,110.59
Endocrine, nutritional and metabolic diseases	3,814.82	5.16	0.72	4,563.24
Congenital abnormality	3,411.68	30.12	0.64	2,732.53
Mental disorder	3,240.54	16.39	0.61	3,731.17
Musculoskeletal and connective tissue diseases	1,187.33	8.01	0.22	1,256.89
Hematopoietic immune diseases	1,126.90	12.46	0.21	1,218.97
Obstetric diseases	380.77	41.76	0.07	450.64
Parasitic disease	221.29	45.89	0.04	168.98
Perinatal diseases	0.00	0.00	0.00	0.00
Dysoemia	10,906.30	6.45	2.05	11,624.61
Other diseases	54,141.92	18.30	10.16	58,091.28

^a^ Note: The underreporting death was corrected for different age groups

## Premature NCD mortality

In 2020, the total probability of dying from cardiovascular diseases, malignant tumors, diabetes and chronic respiratory diseases between 30 and 70 years old in Liangshan was 14.26%. In order of premature NCD mortality, the four NCDs were cardiovascular diseases (7.41%), malignant tumors (4.82%), chronic respiratory diseases (2.17%) and diabetes (0.55%). The total probability of dying from four major NCDs in men was 19.16%, and the premature NCD mortality of cardiovascular diseases was the highest, which was 9.94%. The total probability of dying from four major NCDs in women was 9.27%, and the premature NCD mortality of cardiovascular diseases was also the highest, which was 4.83%. Among the four major NCDs, the probability of dying between 30 and 70 years old in men were higher than those in women (see Table 4).

**Table 4:** Premature NCD mortality in Liangshan in 2020

Death cause	Overall population		Men		Women	
	Number of premature deaths	Premature NCD mortality ^a^ (%)	Number of premature deaths	Premature NCD mortality (%)	Number of premature deaths	Premature NCD mortality (%)
Cardiovascular diseases	1,746	7.41	1,206	9.94	540	4.83
Malignant tumors	1,167	4.82	778	6.49	389	3.26
Chronic respiratory diseases	452	2.17	338	3.33	113	1.05
Diabetes	120	0.55	76	0.70	44	0.41
Total	3,485	14.26	2,398	19.16	1,086	9.27

^a^ Note: The underreporting death was corrected for different age and sex groups

### Life expectancy and cause-eliminated life expectancy

#### Life expectancy

In 2020, the average life expectancy of residents in Liangshan was 76.25 years for the overall population, 72.92 years for men and 80.17 years for women (see Table 6 in Additional file 2).

#### Cause-eliminated life expectancy

**Fig. 7:**
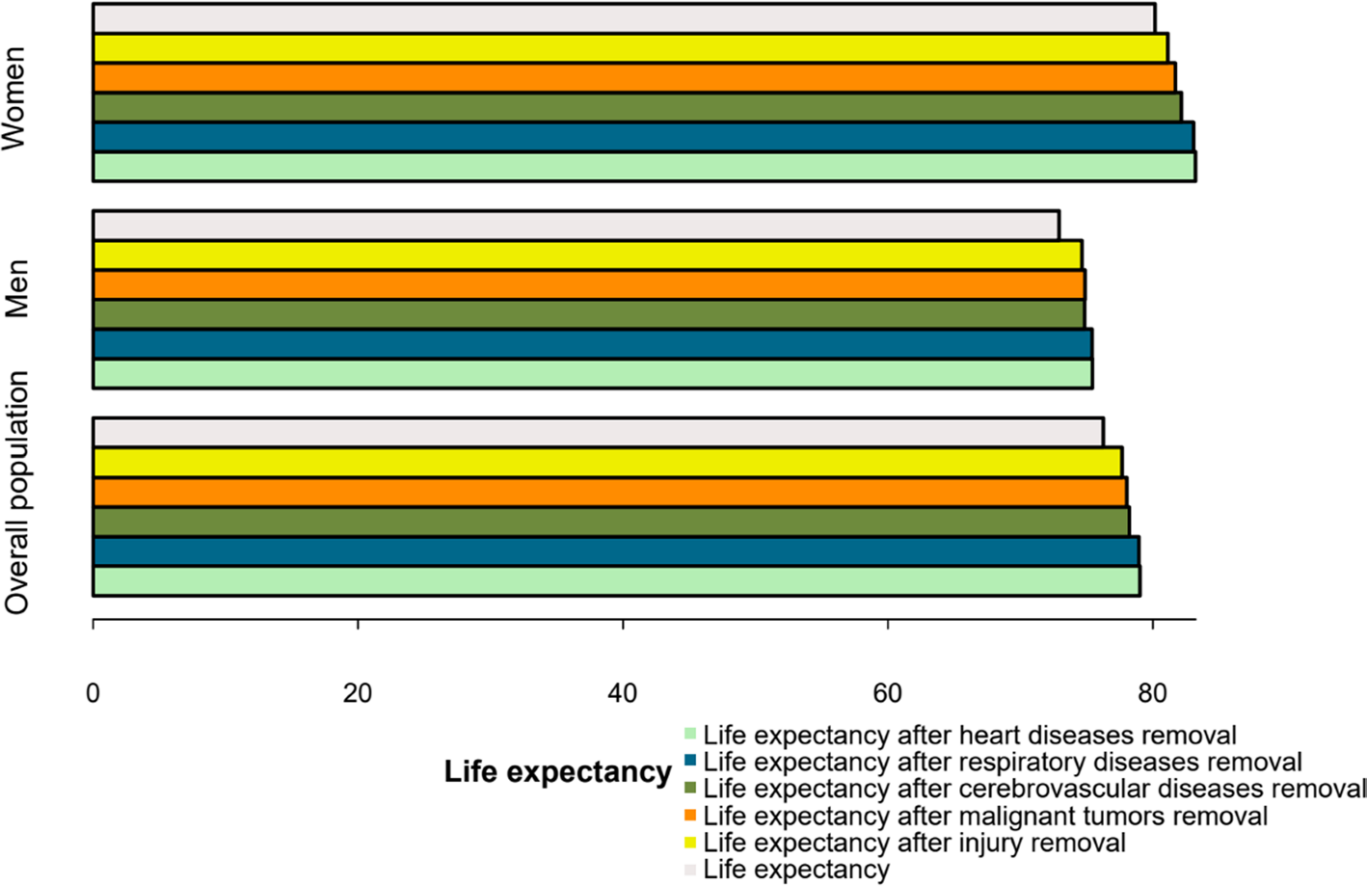
Cause-eliminated life expectancy of different sexes in Liangshan in 2020 (The light green bars represent the life expectancy after heart diseases removal, the blue bars represent the life expectancy after respiratory diseases removal, the olive green bars represent the life expectancy after cerebrovascular diseases removal, the orange bars represent the life expectancy after malignant tumors removal, the yellow bars represent the life expectancy after injury removal, and the light gray bars represent the life expectancy)

After eliminating heart diseases, respiratory diseases, cerebrovascular diseases, malignant tumors and injury, the life expectancy of residents in Liangshan were 79.02 years old, 78.94 years old, 78.22 years old, 78.03 years old and 77.67 years old in turn, and the highest increase in life expectancy was 2.77 years old. In men and women, the life expectancy after eliminating heart diseases were 75.43 and 83.22 years respectively, which were 2.51 and 3.05 years higher than the average life expectancy. After eliminating malignant tumors and injury, the life expectancies increase of men were higher than women’s (Fig. 7).

## Discussion

This study showed the overall mortality rate, age-specific mortality in different gender and economic level counties, death spectrum, life expectancy, cause-eliminated life expectancy and premature NCD mortality in Liangshan in 2020. It was worthwhile to further compare the results of Liangshan one-by-one with other areas, so as to clarify the relative death level of Liangshan and help provide references for the decision-making on health policies in other poverty-stricken areas both at home and abroad.

Mortality rate of residents can reflect the residents’ health level. This study showed that the crude mortality rate of Liangshan residents in 2020 was 608.75/100,000, and the age-standardized death rate was 633.50/100,000.The crude mortality in Liangshan was lower than Sichuan province’s and the whole country’s [17, 18], while after age standardization, the situation reversed [13, 19]. Compared with Vietnam and Laos [20], the mortality in Liangshan was lower, indicating that the mortality rate in Liangshan was in a relative well level in the world’s poverty-stricken areas, so can serve as a reference for other poverty-stricken areas to reduce the mortality rate of residents.

Among different sexes, the crude mortality of men was about 1.47 times of women, consistent with the national results [13], which might be explained by that most men worked under great pressure and were more likely to have bad habits. Among different age groups, the lowest values existed in 5-9 years old groups. The 0-year-old mortality of overall population, men and women were 9.32‰, 8.52‰, and 10.16‰ respectively, which were significantly higher than the level of Sichuan province and the whole country [13, 17]. In addition, the 0-year-old mortality of women was much higher than men, which might be due to some local cultural concepts. These results showed that, consistent with the poverty-stricken areas, such as Vietnam and Laos [21], the health status of infants was poor, while the ability of medical and health services was lacking. We should guide correct social concepts, and at the same time, strengthen health care during pregnancy, childbirth and children. Among the counties with different economic levels, the former severely impoverished counties had lower access to health care opportunities and quality than non-impoverished counties, and their age-standardized mortality, 0-year-old mortality were higher, resulting in serious death status. Therefore, in order to lower the high death level, it is necessary to increase investment in economically backward areas to ensure adequate medical and health resources.

The death spectrum of Liangshan was consistent with most areas at home and abroad, with NCDs as the main cause [22, 23]. This study found that the proportion of NCDs in Liangshan was 75.18%, which was lower than the national level of 88% [24]. At present, the premature NCD mortality of malignant tumors, cardiovascular diseases, chronic respiratory diseases and diabetes is an important indicator to evaluate the control level of NCDs. This study findings indicated that the number of deaths from four major NCDs accounted for 63.07% of the total number of deaths. Besides, the premature mortality of four major NCDs were 14.26% for the overall population, which was lower than that in the whole country, Vietnam and Laos [25], while higher than that in some global areas [22]. The results in the study showed that the mortality of NCDs and the premature mortality of four major NCDs in men were higher than those in women. Previous studies have confirmed smoking, harmful drinking, salt intaking, obesity, elevated blood pressure and blood sugar are risk factors affecting the premature mortality of four major NCDs [26], and exposure to PM_10_ and PM_2.5_ are related to all-cause death, cardiovascular death and respiratory death independently [27]. Therefore, in order to achieve the global Sustainable Development Goals and the Healthy China 2030 Goals [22, 28] earlier and further reduce the premature NCD mortality, relevant departments should vigorously carry out the prevention and health care work of NCDs. When formulating health policies in poverty-stricken areas, men should be regarded as the key population for prevention and control of NCDs, and smoking, drinking, diet and air pollution of local residents should be controlled at the same time, so as to strengthen the publicity of health knowledge and improve the education level of residents.

The top five death causes in Liangshan in 2020 were heart diseases, respiratory diseases, cerebrovascular diseases, malignant tumors and injury, which were consistent with the top five death causes in China in 2019, but the specific rank was slightly different. The mortality rate of heart diseases was lower than the national level, while for respiratory diseases, situation changed oppositely [13]. In the death cause rank, infectious diseases ranked the 7th, with a mortality of 19.94/100,000, which was lower than that in 2018, but still higher than the level of Sichuan province in 2019 [17]. AIDS, tuberculosis, hepatitis and other infectious diseases had high incidence in Liangshan, resulting in high mortality rates,especially for AIDS. Infectious diseases in poverty-stricken areas posed a great threat to the people, such as the high mortality of tuberculosis in Laos, which was 71.41/100,000 [21]. It is necessary to pay attention to the epidemic of infectious diseases in poverty-stricken areas, adopt prevention and control planning measures according to local conditions, control the source of infection, cut off the route of transmission and protect susceptible groups.

Injury, malignant tumors and infectious diseases rose from the 5th, 4th and 7th in the rank of death causes to the first, second and fourth in the rank of life loss, which indicated that the residents with injury, malignant tumors and infectious diseases died at a younger age. Among them, PYLL caused by injury was 1.55 times higher than that in malignant tumors. Meanwhile, due to the aging population, unhealthy diet, bad behavior, environmental pollution and other factors, the incidence of malignant tumors in the world is increasing year by year, and the disease burden is heavy. In addition, PYLLs and SPYLLs caused by various death causes in male residents were higher than those in female residents mostly, indicating men suffer more harm from diseases. Compared with Liangshan, infrastructure in countries like Laos is even less developed, which brings potential health hazards that deserve attention. Therefore, the relevant departments in poverty-stricken areas should form a multi-sectoral cooperation mechanism, pay attention to male health problems, carry out etiological prevention, and take some comprehensive measures to control the serious health burden caused by injury, tumors and infectious diseases.

Life expectancy can reflect the health level of residents comprehensively, while cause-eliminated life expectancy can reflect the loss of life expectancy caused by a specific disease. In 2020, the life expectancy was 76.25 years for Liangshan residents, which was lower than the national level, while higher than the global, Vietnam and Laos [25]. After eliminating heart diseases, life expectancy has increased by 2.77 years. After eliminating heart diseases, respiratory diseases and cerebrovascular diseases, women’s life expectancy increased more than men’s, indicating that these three diseases had a greater impact on women.

This study revealed the latest death status in Liangshan, and indicated Liangshan might become a well reference for reducing deaths in poverty-stricken areas. However, some improvements are still required. First, Liangshan was not declared out of poverty until 2020. Second, data of the investigation on underreporting deaths for 2020 in some areas have not yet been updated. Therefore, to obtain more sample sizes and improve the accuracy and representativeness of the results, it is necessary to keep close attention to death surveillance in Liangshan for a long time in the future. In spite of this, the study of the death situation in Liangshan can not only promote our understanding of the current death situation in poverty-stricken areas, but also may shed light on more solutions to the possible impact of economic development on death, and thus improve the life expectancy of people in similar areas both at home and abroad to promote global health.

## Conclusions

This study suggested we should focus on the death situation in poverty-stricken areas, especially in economically backward areas, strengthen women’s and children’s health care, pay attention to men’s health, lower the death level of NCDs and infectious diseases, reduce the adverse health outcomes caused by injuries, formulate medical policies rationally, raise the diagnosis and treatment level of medical institutions, improve residents’ health literacy, and realize *healthy China* and *healthy world* as soon as possible. The results of this study also provide ideas for further exploring the solutions to the possible impact of economic development on death, and thus improve the life expectancy of people living in poverty-stricken areas in the world.

## Supplementary Information

**Additional file 1.** Calculation method of death-related indicators. This Additional file is an introduction to the method of analyzing death-related indicators in this study.

**Additional file 2.** This Additional file contains six tables, which present some results of this study.

## Data Availability

According to the regulation of data management, the data of this study could be made available by applying to the corresponding author who is on behalf of the research team.

## Abbreviations

Liangshan: Liangshan Yi Autonomous Prefecture
NCDs: Noncommunicable diseases
CRC: Capture-recapture
PYLL: Potential years of life lost
AYLL: Average years of life lost
PYLLR: Potential years of life lost rate
SPYLL: Standardized potential years of life lost
AIDS: Acquired Immune Deficiency Syndrome
