# Supplementary material for: Analysis of death causes of residents in poverty-stricken Areas in 2020: take Liangshan Yi Autonomous Prefecture in China as an example
Source: BMC Public Health. 2022 Jan 13;22:89. doi: 10.1186/s12889-022-12504-6 (PMC8758188; doi:10.1186/s12889-022-12504-6)
Supplement: Supplementary file 1 — Additional file 1. Calculation method of death-related indicators. This Additional file is an introduction to the method of analyzing death-related indicators in this study. [file 12889_2022_12504_MOESM1_ESM.docx]

**Additional file 1**

**Calculation method of death-related indicators**

- **Mortality rate** [1]

Mortality rate is the crude mortality rate, which can reflect the death situation of the population.

$$\text{mortality rate}=\frac{\text{the number of deaths }}{\text{the number of population}}\times100000/100000$$

In the sex-specific mortality rate and age-specific mortality rate, the corresponding number of deaths are the number of deaths of a certain sex and a certain age respectively, and the corresponding number of the population is the number of the population of a certain gender and a certain age group. In the cause-specific mortality rate, the corresponding number of deaths is the number of deaths due to a particular cause, and the number of the population is the same as that at the time of calculating mortality.

- **Age-standardized mortality** [2]

Age-standardized mortality is the mortality calculated by using the standard population proportion and the actual age-specific mortality, which is used to compare the whole population mortality in two or more areas with different age structures. In this study, the standard population proportion data of China's sixth census in 2010 were used to standardize the mortality rate.

- **Potential years of life lost (PYLL)** [3]

Potential years of life lost refers to the sum of the difference between life expectancy and actual death age of people in a certain age group due to a certain disease, that is, the life loss caused by death. It is an index used to measure the burden of diseases, and it can assess the harm of residents’ premature death due to diseases to the health of people.

$$PYLL=\sum A_{i}\times D_{i}=\sum(L-X-0.5)\times D_{i}$$

In the formula, *A*-remaining age, *D*- number of deaths in a certain age group, *L*- life expectancy (years), and *X*- median of each age group. In this study, the potential years of life lost were calculated with 70 years as the target survival age standard, and the influence of 0-year-old group and high age group on life loss were eliminated.

- **Average years of life lost (AYLL)** [4]

Average years of life lost refers to the average value of the difference between life expectancy and actual age of death, reflecting the average life loss of each deceased.

$$AYLL=\frac{PYLL}{D}$$

In the formula, *D*- the total number of deaths from a cause of death in the same period.

- **Potential years of life lost rate (PYLLR)**

Potential years of life lost rate refers to the ratio of potential years of life lost to the total population of a certain population in the same period, reflecting the harm degree of different death causes to different populations.

$$PYLLR=\frac{PYLL}{N}\times1000\text{‰}$$

In the formula, *N*- the total population of a certain population in the same period.

- **Standardized potential years of life lost (SPYLL)**

Standardized potential years of life lost is an index of standardizing potential years of life lost to eliminate the impact of population structure.

$$SPYLL=\sum PYLL_{X}\times C_{X}$$

$$C_{X}=\frac{\text{standard age group population/total standard population}}{\text{current population of age group/total current population}}$$

In the formulas, *PYLL_x_*- years of life loss of age group *x*, *C_X_*- correction coefficient of age group *x*.

- **Premature NCD mortality** [5]

Premature NCD mortality refers to the probability of dying from four NCDs between 30 to 70 years old, is a probability indicator calculated based on the life table method. The probability of dying from cardiovascular diseases, malignant tumors, diabetes and chronic respiratory diseases among people aged 30-70 is calculated, which means the probability of dying from these four NCDs before the 70th birthday of the 30-year-old people in a certain year.

$${}_{5}M_{X}=\frac{\text{the number of deaths in a certain age group}}{\text{the total number of a certain age group}}$$

$${}_{5}q_{X}=\frac{{}_{5}M_{X}\times5}{1+{}_{5}M_{X}\times2.5}$$

$$_{40}q_{30}=1-\prod_{x-30}^{69} (1-_{5}q_{x})$$

In the formulas, _5_*M_x_*- five-year mortality rate of an age group, _5_*q_x_*- death probability of an age group.

- **Life expectancy** [6]

Life expectancy refers to the average life expectancy of a generation born at the same time when they live to the age of *X*, that is, the average number of years that *X*-year-old survivors may continue to live under a certain death level. In this study, a brief life table was compiled to calculate the life expectancy at birth.

$$e_{X}=\frac{T_{X}}{l_{X}}$$

In the formula, *e_x_*- life expectancy, *T_x_*- total number of survival person-years, *l_x_*- number of survivors.

- **Cause eliminated life expectancy** [2]

Cause eliminated life expectancy is to calculate the life expectancy of people after the death caused by a certain disease is eliminated, and the calculation method is similar to life expectancy. It can reflect the harm of certain diseases to people's health, and comprehensively evaluate the effect of a certain cause of death on the whole population in a certain area.

- **Corrected mortality rate**

By using the capture-recapture method, the underreporting rates of death of the overall population, men and women in different age groups, the underreporting rates of death of former severely impoverished counties and non-impoverished counties in different age groups were calculated, and all death related indicators were corrected.

$$\text{corrected mortality rate}=\frac{\text{mortality rate}}{\text{1-0.01}*\text{R}}$$

In the formula, R- the underreporting rates of death.

**References**

1. Center for Chronic Non-communicable Disease Prevention and Control, Chinese Center for Disease Control and Prevention, Statistical Information Center of National Health Commission. *China Death Surveillance Data Set 2019*. Beijing: China Science and Technology Press; 2020.
2. Kunst AE, Amiri M, Janssen F. The decline in stroke mortality: exploration of future trends in 7 Western European countries. *Stroke*. 2011;42(8):2126-2130.
3. Islami F, Miller KD, Siegel RL, Zheng Z, Zhao J, Han X, et al. National and state estimates of lost earnings from cancer deaths in the United States. *JAMA Oncol*. 2019;5(9):e191460.
4. Ludwig H, Bolejack V, Crowley J, Bladé J, Miguel JS, Kyle RA, et al. Survival and years of life lost in different age cohorts of patients with multiple myeloma. *J Clin Oncol*. 2010;28(9):1599-1605.
5. World Health Organization (WHO). Global non-communicable diseases 2014. <http://www.who.int/nmh/publications/ncd-status-report-2014/en/>. Accessed 13 July 2021.
6. Gross CP, McAvay GJ, Krumholz HM, Paltiel AD, Bhasin D, Tinetti ME. The effect of age and chronic illness on life expectancy after a diagnosis of colorectal cancer: implications for screening. *Ann Intern Med*. 2006;145(9):646-653.
