# Supplementary material for: Analysis of death causes of residents in poverty-stricken Areas in 2020: take Liangshan Yi Autonomous Prefecture in China as an example
Source: BMC Public Health. 2022 Jan 13;22:89. doi: 10.1186/s12889-022-12504-6 (PMC8758188; doi:10.1186/s12889-022-12504-6)
Supplement: Supplementary file 2 — Additional file 2. This Additional file contains six tables, which present some results of this study. [file 12889_2022_12504_MOESM2_ESM.docx]

**Additional file 2**

Table 1 Mortality rate of Liangshan residents by sex and age in 2020 (per 100,000)

| Age (years) | Overall population | | | Men | | | Women | | |
| --- | --- | --- | --- | --- | --- | --- | --- | --- | --- |
|  | Number of deaths | Proportion (%) | Mortality rate | Number of deaths | Proportion (%) | Mortality rate | Number of deaths | Proportion (%) | Mortality rate |
| 0 | 176 | 1.04 | 931.75 | 101 | 0.99 | 851.59 | 74 | 1.12 | 1,016.18 |
| 1—4 | 147 | 0.87 | 122.51 | 84 | 0.82 | 112.26 | 63 | 0.95 | 129.10 |
| 5—9 | 95 | 0.56 | 31.63 | 64 | 0.63 | 45.94 | 31 | 0.47 | 17.44 |
| 10—14 | 109 | 0.65 | 59.12 | 74 | 0.72 | 66.67 | 35 | 0.53 | 45.40 |
| 15—19 | 141 | 0.84 | 68.42 | 85 | 0.83 | 91.96 | 56 | 0.85 | 40.56 |
| 20—24 | 159 | 0.94 | 73.46 | 113 | 1.10 | 97.51 | 46 | 0.70 | 44.33 |
| 25—29 | 230 | 1.36 | 138.24 | 183 | 1.79 | 190.11 | 47 | 0.71 | 77.21 |
| 30—34 | 338 | 2.01 | 262.50 | 265 | 2.59 | 350.96 | 72 | 1.09 | 163.50 |
| 35—39 | 466 | 2.77 | 375.86 | 366 | 3.57 | 568.74 | 100 | 1.52 | 158.65 |
| 40—44 | 554 | 3.29 | 358.11 | 428 | 4.18 | 534.21 | 126 | 1.91 | 170.41 |
| 45—49 | 794 | 4.71 | 376.59 | 616 | 6.02 | 572.94 | 178 | 2.70 | 169.77 |
| 50—54 | 1,005 | 5.96 | 537.04 | 745 | 7.28 | 728.42 | 260 | 3.94 | 356.57 |
| 55—59 | 821 | 4.87 | 627.61 | 568 | 5.55 | 853.48 | 253 | 3.83 | 399.07 |
| 60—64 | 829 | 4.92 | 1,002.12 | 554 | 5.41 | 1,373.51 | 275 | 4.17 | 642.40 |
| 65—69 | 1,413 | 8.39 | 1,696.61 | 909 | 8.88 | 2,434.56 | 504 | 7.64 | 1,048.63 |
| 70—74 | 1,850 | 10.98 | 2,551.83 | 1,087 | 10.62 | 3,219.50 | 760 | 11.52 | 1,919.85 |
| 75—79 | 2,473 | 14.68 | 4,698.18 | 1,393 | 13.59 | 5,505.51 | 1,076 | 16.31 | 3,956.54 |
| 80—84 | 2,611 | 15.50 | 8,146.17 | 1,388 | 13.56 | 9,489.25 | 1,221 | 18.51 | 6,990.53 |
| 85—89 | 1,678 | 9.96 | 11,545.45 | 805 | 7.86 | 12,367.64 | 871 | 13.20 | 10,839.96 |
| 90—94 | 762 | 4.52 | 19,548.72 | 324 | 3.16 | 20,070.11 | 438 | 6.64 | 18,710.83 |
| 95—99 | 171 | 1.01 | 23,427.02 | 77 | 0.75 | 25,341.95 | 94 | 1.42 | 21,342.36 |
| ≥ 100 | 28 | 0.17 | 34,324.18 | 10 | 0.10 | 27,455.32 | 18 | 0.27 | 31,528.36 |
| Total | 16,850^a^ | 100.00 | 608.75 | 10,239 | 100.00 | 720.60 | 6,598 | 100.00 | 489.26 |

^a^Note: The sexes of the 13 death cases were unknown, and they were excluded when calculating by sex.

Table 2 Mortality rate of Liangshan residents by economic level and age in 2020

(per 100,000)

| Age (years) | Former severely impoverished counties | | | Non-impoverished counties | | |
| --- | --- | --- | --- | --- | --- | --- |
|  | Number of deaths | Proportion (%) | Mortality rate | Number of deaths | Proportion (%) | Mortality rate |
| 0 | 82 | 2.51 | 1,290.52 | 94 | 0.69 | 351.66 |
| 1—4 | 70 | 2.15 | 151.36 | 77 | 0.57 | 70.39 |
| 5—9 | 46 | 1.41 | 42.38 | 49 | 0.36 | 21.06 |
| 10—14 | 48 | 1.47 | 78.73 | 61 | 0.45 | 44.58 |
| 15—19 | 71 | 2.18 | 99.90 | 70 | 0.52 | 48.09 |
| 20—24 | 56 | 1.72 | 96.65 | 102 | 0.75 | 56.15 |
| 25—29 | 96 | 2.94 | 272.09 | 134 | 0.99 | 75.80 |
| 30—34 | 163 | 5.00 | 478.62 | 175 | 1.29 | 140.23 |
| 35—39 | 208 | 6.38 | 628.49 | 258 | 1.90 | 188.79 |
| 40—44 | 217 | 6.65 | 651.31 | 337 | 2.48 | 238.73 |
| 45—49 | 197 | 6.04 | 637.10 | 597 | 4.40 | 305.15 |
| 50—54 | 230 | 7.05 | 890.81 | 775 | 5.71 | 437.37 |
| 55—59 | 162 | 4.97 | 682.61 | 658 | 4.84 | 561.31 |
| 60—64 | 157 | 4.81 | 1,224.07 | 672 | 4.95 | 939.07 |
| 65—69 | 237 | 7.27 | 2,337.36 | 1,175 | 8.65 | 1,507.47 |
| 70—74 | 311 | 9.53 | 2,697.23 | 1,539 | 11.33 | 2,482.97 |
| 75—79 | 371 | 11.37 | 5,386.33 | 2,102 | 15.48 | 4,508.31 |
| 80—84 | 312 | 9.56 | 9,255.88 | 2,298 | 16.90 | 8,015.11 |
| 85—89 | 158 | 4.84 | 11,346.81 | 1,520 | 11.19 | 11,678.95 |
| 90—94 | 56 | 1.72 | 22,616.15 | 705 | 5.19 | 17,838.33 |
| 95—99 | 11 | 0.34 | 14,187.94 | 160 | 1.18 | 20,213.22 |
| ≥ 100 | 3 | 0.09 | 18,333.59 | 25 | 0.18 | 26,945.43 |
| Total | 3,262 | 100.00 | 585.82 | 13,583 | 100.00 | 617.36 |

Note: The counties where the 5 death cases lived before death did not belong to Liangshan, and they were eliminated.

Table 3 Mortality and proportion of three categories of diseases in Liangshan in 2020

| Category of diseases | Overall population | | | Men | | | Women | | |
| --- | --- | --- | --- | --- | --- | --- | --- | --- | --- |
|  | Number of deaths | Proportion (%) | Mortality rate (per 100,000) | Number of deaths | Proportion (%) | Mortality rate (per 100,000) | Number of deaths | Proportion (%) | Mortality rate (per 100,000) |
| The first category of diseases | 1,230 | 7.30 | 44.44 | 783 | 7.65 | 55.11 | 444 | 6.73 | 32.92 |
| The second category of diseases | 12,668 | 75.18 | 457.66 | 7,513 | 73.37 | 528.75 | 5,147 | 78.01 | 381.67 |
| The third category of diseases | 1,215 | 7.21 | 43.89 | 895 | 8.74 | 62.99 | 319 | 4.83 | 23.65 |
| Other diseases | 1,737 | 10.31 | 62.75 | 1,048 | 10.24 | 73.76 | 688 | 10.43 | 51.02 |

Note: The first category of diseases refers to infectious diseases, maternal-infant diseases and nutritional deficiency diseases, the second category of diseases refers to chronic non-communicable diseases, and the third category of diseases refers to injury.

Table 4 Mortality rate of death causes in Liangshan in 2020 (per 100,000)

| Rank of death cause | Overall population | | | | Men | | | | Women | | | |
| --- | --- | --- | --- | --- | --- | --- | --- | --- | --- | --- | --- | --- |
|  | Disease | Mortality rate | Standardized mortality rate | Proportion (%) | Disease | Mortality rate | Standardized mortality rate | Proportion (%) | Disease | Mortality rate | Standardized mortality rate | Proportion (%) |
| 1 | Heart diseases | 112.07 | 109.94 | 18.41 | Heart diseases | 120.49 | 119.55 | 16.72 | Heart diseases | 102.92 | 99.24 | 21.05 |
| 2 | Respiratory diseases | 105.85 | 101.80 | 17.39 | Respiratory diseases | 119.01 | 114.95 | 16.52 | Respiratory diseases | 91.43 | 87.69 | 18.69 |
| 3 | Cerebrovascular diseases | 87.03 | 87.04 | 14.30 | Cerebrovascular diseases | 97.83 | 100.31 | 13.58 | Cerebrovascular diseases | 75.41 | 73.19 | 15.41 |
| 4 | Malignant tumors | 73.92 | 80.39 | 12.14 | Malignant tumors | 93.53 | 103.23 | 12.98 | Malignant tumors | 53.17 | 57.84 | 10.87 |
| 5 | Injury | 43.89 | 50.57 | 7.21 | Injury | 62.99 | 72.23 | 8.74 | Injury | 23.65 | 26.71 | 4.83 |
| 6 | Digestive system diseases | 33.45 | 37.06 | 5.50 | Digestive system diseases | 46.94 | 53.41 | 6.51 | Digestive system diseases | 19.21 | 19.84 | 3.93 |
| 7 | Infectious diseases | 19.94 | 25.16 | 3.28 | Infectious diseases | 26.95 | 33.96 | 3.74 | Endocrine, nutritional and metabolic diseases | 13.35 | 13.10 | 2.73 |
| 8 | Endocrine, nutritional and metabolic diseases | 14.34 | 14.44 | 2.36 | Urogenital system diseases | 15.55 | 15.56 | 2.16 | Infectious diseases | 12.53 | 15.64 | 2.56 |
| 9 | Urogenital system diseases | 12.50 | 12.56 | 2.05 | Endocrine, nutritional and metabolic diseases | 15.27 | 15.75 | 2.12 | Urogenital system diseases | 9.27 | 9.36 | 1.89 |
| 10 | Nervous system diseases | 8.53 | 8.91 | 1.40 | Nervous system diseases | 9.92 | 10.39 | 1.38 | Nervous system diseases | 7.04 | 7.39 | 1.44 |
| 11 | Mental disorder | 3.54 | 4.00 | 0.58 | Mental disorder | 4.57 | 5.21 | 0.63 | Musculoskeletal and connective tissue diseases | 2.97 | 3.10 | 0.61 |
| 12 | Musculoskeletal and connective tissue diseases | 2.85 | 2.88 | 0.47 | Perinatal diseases | 2.89 | 3.78 | 0.40 | Mental disorder | 2.37 | 2.56 | 0.48 |
| 13 | Perinatal diseases | 2.46 | 3.72 | 0.40 | Musculoskeletal and connective tissue diseases | 2.74 | 2.64 | 0.38 | Perinatal diseases | 1.93 | 3.47 | 0.39 |
| 14 | Hematopoietic immune diseases | 1.66 | 1.76 | 0.27 | Hematopoietic immune diseases | 2.04 | 2.11 | 0.28 | Congenital abnormality | 1.33 | 1.66 | 0.27 |
| 15 | Congenital abnormality | 1.59 | 2.02 | 0.26 | Congenital abnormality | 1.83 | 2.22 | 0.25 | Hematopoietic immune diseases | 1.26 | 1.35 | 0.26 |
| 16 | Obstetric diseases | 0.14 | 0.21 | 0.02 | Parasitic diseases | 0.14 | 0.16 | 0.02 | Obstetric diseases | 0.30 | 0.46 | 0.06 |
| 17 | Parasitic disease | 0.07 | 0.09 | 0.01 | Obstetric diseases | 0.00 | 0.00 | 0.00 | Parasitic diseases | 0.00 | 0.00 | 0.00 |
| - | Dysoemia | 31.94 | 31.05 | 5.25 | Dysoemia | 33.08 | 32.40 | 4.59 | Dysoemia | 30.70 | 29.23 | 6.27 |
| - | Other diseases | 52.96 | 59.91 | 8.70 | Other diseases | 64.82 | 74.06 | 9.00 | Other diseases | 40.41 | 45.21 | 8.26 |

Table 5 Analysis of potential life loss of Liangshan residents by sex in 2020

| Rank of life loss | | Men | | | | | Women | | | |  |
| --- | --- | --- | --- | --- | --- | --- | --- | --- | --- | --- | --- |
|  | PYLL  (person years) | | AYLL (years/person) | PYLLR  (‰) | SPYLL  (person years) | Death cause | PYLL  (person years) | AYLL (years/person) | PYLLR  (‰) | SPYLL  (person years) | Death cause |
| 1 | 48,647.99 | | 26.48 | 17.75 | 49,797.10 | Injury | 14,451.34 | 21.17 | 5.58 | 13,593.68 | Injury |
| 2 | 27,562.80 | | 10.87 | 10.06 | 31,468.97 | Malignant tumors | 14,338.16 | 10.22 | 5.53 | 16,165.46 | Malignant tumors |
| 3 | 21,461.25 | | 26.09 | 7.83 | 23,546.16 | Infectious diseases | 8,983.23 | 3.85 | 3.47 | 7,780.68 | Respiratory diseases |
| 4 | 20,940.35 | | 6.52 | 7.64 | 24,490.56 | Heart diseases | 8,729.88 | 3.39 | 3.37 | 9,422.59 | Heart diseases |
| 5 | 18,932.92 | | 7.24 | 6.91 | 22,462.58 | Cerebrovascular diseases | 7,806.32 | 4.11 | 3.01 | 8,550.61 | Cerebrovascular diseases |
| 6 | 15,806.39 | | 12.29 | 5.77 | 19,421.32 | Digestive system diseases | 7,600.57 | 20.50 | 2.93 | 8,350.12 | Infectious diseases |
| 7 | 14,483.89 | | 4.59 | 5.29 | 16,223.77 | Respiratory diseases | 3,150.79 | 6.42 | 1.22 | 3,774.11 | Digestive system diseases |
| 8 | 4,237.93 | | 15.33 | 1.55 | 4,122.90 | Nervous system diseases | 2,514.96 | 13.31 | 0.97 | 2,444.82 | Nervous system diseases |
| 9 | 2,846.33 | | 6.86 | 1.04 | 3,258.46 | Urogenital system diseases | 1,665.00 | 33.98 | 0.64 | 1,223.50 | Congenital abnormality |
| 10 | 2,545.71 | | 6.28 | 0.93 | 3,137.22 | Endocrine, nutritional and metabolic diseases | 1,583.96 | 6.69 | 0.61 | 1,789.86 | Urogenital system diseases |
| 11 | 2,422.31 | | 18.62 | 0.88 | 2,808.17 | Mental disorder | 1,330.76 | 3.98 | 0.51 | 1,504.10 | Endocrine, nutritional and metabolic diseases |
| 12 | 1,641.05 | | 27.02 | 0.60 | 1,398.53 | Congenital abnormality | 801.59 | 12.57 | 0.31 | 867.54 | Mental disorder |
| 13 | 676.81 | | 9.13 | 0.25 | 657.55 | Musculoskeletal and connective tissue diseases | 495.62 | 15.12 | 0.19 | 531.71 | Hematopoietic immune diseases |
| 14 | 585.34 | | 10.22 | 0.21 | 638.83 | Hematopoietic immune diseases | 472.31 | 6.45 | 0.18 | 578.04 | Musculoskeletal and connective tissue diseases |
| 15 | 188.99 | | 43.92 | 0.07 | 151.81 | Parasitic diseases | 400.47 | 41.93 | 0.15 | 489.14 | Obstetric diseases |
| 16 | 0.00 | | 0.00 | 0.00 | 0.00 | Perinatal diseases | 0.00 | 0.00 | 0.00 | 0.00 | Parasitic diseases |
| 17 | 0.00 | | 0.00 | 0.00 | 0.00 | Obstetric diseases | 0.00 | 0.00 | 0.00 | 0.00 | Perinatal period |
| — | 8,398.59 | | 9.26 | 3.07 | 9,034.06 | Dysoemia | 2,199.97 | 2.85 | 0.85 | 2,299.62 | Dysoemia |
| — | 35,412.98 | | 19.16 | 12.92 | 38,667.02 | Other diseases | 18,822.56 | 16.83 | 7.26 | 19,251.54 | Other diseases |

Table 6 Influence of main death causes on life expectancy of residents in Liangshan in 2020

| Death cause | Overall population | | Men | | Women | |
| --- | --- | --- | --- | --- | --- | --- |
|  | Cause-eliminated life expectancy (years) | Life expectancy increases (years) | Cause-eliminated life expectancy (years) | Life expectancy increases (years) | Cause-eliminated life expectancy (years) | Life expectancy increases (years) |
| Heart diseases | 79.02 | 2.77 | 75.43 | 2.51 | 83.22 | 3.05 |
| Respiratory diseases | 78.94 | 2.69 | 75.41 | 2.49 | 83.06 | 2.89 |
| Cerebrovascular diseases | 78.22 | 1.97 | 74.84 | 1.92 | 82.15 | 1.98 |
| Malignant tumors | 78.03 | 1.78 | 74.87 | 1.95 | 81.69 | 1.52 |
| Injury | 77.67 | 1.42 | 74.65 | 1.73 | 81.13 | 0.96 |
